# Who Makes Your Heart Beat? What Makes You Sweat? Social Conflict in Virtual Reality for Educators

**DOI:** 10.3389/fpsyg.2021.628246

**Published:** 2021-05-28

**Authors:** Minha Lee, Jan Kolkmeier, Dirk Heylen, Wijnand IJsselsteijn

**Affiliations:** ^1^Future Everyday, Department of Industrial Design, Eindhoven University of Technology, Eindhoven, Netherlands; ^2^Human Media Interaction, University of Twente, Enschede, Netherlands; ^3^Human Technology Interaction, Department of Industrial Engineering and Innovation Sciences, Eindhoven University of Technology, Eindhoven, Netherlands

**Keywords:** VR training, physiology, social conflict, education, cultural comparison

## Abstract

Though educators often deal with stressful social conflicts, many face them *ad hoc* without much training. We studied if and how virtual agents can help University staff manage student-teacher conflicts. We explored educators' verbal, behavioral, and physiological reactions to a virtual agent that brought up a student-teacher conflict and held exit-interviews. Our qualitative analysis revealed that virtual agents for conflict training were positively received, but not for conflict mediation with cross-cultural differences. Those with non-Western backgrounds felt that an agent could help “save face,” whereas Westerners preferred to resolve conflicts in person. In line with this, participants with a Western background rated the virtual agent to be less competent compared to those with non-Western backgrounds. While physiological measures only allow for limited conclusions, we found that participants who believed that the agent was controlled by a human had higher normalized hear rate variability (for the entire conversation in total) than people who thought that the agent was autonomous. We discuss implications for implementing virtual agents for training purposes, the impact of physiological signals, and the need to consider cultural and individual differences.

## 1. Introduction

Student-teacher social conflicts can be complex. For one, grade-related conflicts are notoriously common in higher education, causing perennial strifes between students and University teaching staff (Roosevelt, 2009). Educators may view students as acting unduly entitled to higher grades based on their perceived effort rather than achievement (Roosevelt, 2009), but students may feel that the faculty have an upper hand in power dynamics, with instructors acting authoritatively (Jamieson and Thomas, 1974). Managing student-teacher conflicts can involve institutional mediation. Yet, the issue is that educators are often unprepared to face common conflicts like grade disputes until they experience them in real life, which is the case for many work-related conflicts.

VR has helped people undergo training not only in the domain of education, but in medical and psychotherapy domains (Hoffman and Vu, 1997; Glanz et al., 2003; Pantelidis, 2010; Ke et al., 2016). For training purposes, one's sense of presence that VR induces is an important factor, not VR in itself (Grassini et al., 2020). The strength of VR is in experiential realism for delivering educational material (Helsel, 1992; Mikropoulos and Natsis, 2011). VR hence has been used more for educational purposes, e.g., one can learn cultural customs alongside a foreign language (Cheng et al., 2017), than for training educators themselves. Conflict management training is one specific interaction that educators can potentially benefit from.

Though virtual reality (VR) has been heralded as a promising avenue for analyzing and mediating conflicts (Shufutinsky et al., 2018), VR conflict management research is scarce. A few examples are for conflicts that arise in surgeries (Robb et al., 2015), police training (Bruijnes et al., 2015), or inter-group tension (Hasler et al., 2014). There has been no focus on teaching professionals' dyadic, social conflict experiences [a short work on interpersonal gaze is an exception (Kolkmeier et al., 2017b)]. What is missing in prior VR research is considering social conflicts as one-on-one *conversations* in which one's culture plays a critical part in assessing a virtual trainer.

In our study, the goal was to understand conversational social conflict holistically. We did this through triangulating qualitative and quantitative data. With qualitative data, we got participants' first-person report of how the virtual agent made them feel and whether they would use such a system for training. In doing so, we were attentive to participants' cross-cultural differences. Supporting this aim, we added physiological (heart rate and heart rate variability) measurements because social conflicts involve people's physiological and emotional responses (Hardy and Smith, 1988; Kreibig, 2010). There is research on physiological responses to environmental stressors in VR, e.g., threatening scene (Meehan et al., 2002; Garau et al., 2005; Blankendaal et al., 2015), but there is less focus on how people react to a VR agent's conversations. We put together educators' perception of a VR agent during a difficult conversation on student-teacher conflicts, their self-reported accounts of how the virtual agent made them feel, and quantitatively measured physiological states. Broadly, our study provides building blocks for research on culturally appropriate VR agents in work contexts while accounting for educators' physiological signatures. To start, we provide a background in the next section, followed by methods employed, our results, and implications of the work.

## 2. Literature Review

We first define what social conflict is, consider how it can be stressful for educators, and then discuss relevant research on virtual social conflicts.

### 2.1. What Is a Social Conflict?

Broadly, a definition of a social conflict is *a process in which there is a perceived mismatch between one's and another person's (or people's) beliefs or interests* (De Dreu et al., 2004). Social conflicts also are influenced by involved actors' age and culture, as well as their domain of work. For instance, a cross-cultural comparison showed that interpersonal conflicts were significantly distressing for the Japanese, but not for Americans; interpersonal friction, i.e., the lack of assertive behavior leading to avoidance, was distressing for both Americans and Japanese, though frictions were more frequent in Japan (Hashimoto et al., 2012). But, cultural differences in physiological responsiveness are not entirely clear, e.g., between Asians and Non-Asians in interpersonal conflicts (Tsai and Levenson, 1997; Tsai et al., 2002). How people manage (or avoid) social conflicts depend on interpersonal differences like culture, made more complex by their domain of work.

### 2.2. Are Social Conflicts Stressful?

Educators handle many types of conflicts (Findlen, 2000) with a common type being grading conflicts with students at universities who may have different views on, e.g., fair grading (Carless, 2006). Minor conflicts can be stressful short-term and may lead to negative health outcomes unless managed properly (De Dreu et al., 2004), especially for inexperienced teaching staff. To add, educators are often overburdened by teaching duties (Curtis, 2002), which affects their health. In one study, the number of students predicted burnout, with younger University staff more susceptible to emotional exhaustion (Watts and Robertson, 2011). Teaching tasks (e.g., supervising students, long teaching hours) and related stressors are associated with health risks like exhaustion and issues with colleagues can bring about psychological withdrawal (e.g., reduced commitment to one's job) (Taris et al., 2001). Indeed, when unresolved, conflicts (or frictions) can induce stress, with detrimental outcomes on people's well-being (Bolger et al., 1989; Appelberg et al., 1996; Friedman et al., 2000; De Dreu et al., 2004).

Everyday stressors like conflicts with students or too many work priorities can compound, signaling psychological stress that are intertwined with physiological signatures: heart rate (HR), heart rate variability (HRV) as HR consistency over time, and electrodermal activity (EDA) that measures activation of sweat glands, consisting of “resting” tonic skin conductance level (SCL) and “peak” phasic skin conductance response (SCR) (McEwen and Lasley, 2002). Though our “fight-or-flight” response is a natural and healthy means of maintaining balance short-term, we often accumulate everyday stress that foreshadows physical and mental problems that endanger our health (Selye, 1956; Sapolsky, 1994; McEwen and Lasley, 2002). In general, physiological signatures can indicate how people are emotionally affected in stress-inducing situations (Fooken and Schaffner, 2016). Yet, each individual processes stress differently.

People attribute different meanings to situational variables (Lazarus et al., 1985; Lazarus, 2006), which leads individuals to experience and display stress in divergent ways (Lazarus, 1993; Denson et al., 2009). Classically, stress is said to impact us in three phases, (1) alarm (“fight-or-flight”), when the sympathetic nervous system reacts to threats, (2) adaptation, when pituitary glands re-balance, and (3) exhaustion as resource depletion, yet there are interpersonal differences (Selye, 1956). Also, perceived physiological arousal vs. actual arousal can differ (Bosse et al., 2018; Lucas et al., 2018). Thus, social conflicts may bring about different types of emotions and physiological reactions in people. For instance, certain emotions increase HR (e.g., anger, anxiety, anticipatory sadness, joy) while HR decreases for other emotions, e.g., suspense, fear, or sadness (Kreibig, 2010). Some may feel fear during social conflicts while others confront it with anger, which would show different HR changes.

### 2.3. Are Virtual Social Conflicts Stressful?

VR scenarios can be stressful, yet physiological reactions in VR vary per activity. Stress induction tasks (e.g., *ad hoc* presentations) in VR and real-life evoked similar levels of physiological stress, save for cortisol levels which were higher for real-life tasks (Shiban et al., 2016). A threatening VR environment can induce presence (the feeling of “being there”) with an increase in HR as a more reliable and consistent measure across participants than EDA (Meehan et al., 2002). EDA increased in immersive VR with VAs, but HR was significantly greater only when VAs were socially responsive (e.g., eye gaze toward the user) rather than static (e.g., still, or no engagement) (Garau et al., 2005). Thus, a VA's behavior influences certain physiological signals differently.

People have different expectations on virtual trainers compared to human trainers. In a fitness scenario (cycling), people responded that they had better rapport and put in more effort with a positive virtual trainer that used messages like “good job!” But, they actually performed more intensely (as measured by HR) with a negative virtual trainer who gave feedback like “get moving!” A negative agent was not well-favored, but a human trainer that used negative feedback was well-received (Lucas et al., 2018). In another study, a VR cycling scene promoted enjoyment, but a virtual coach did not increase people's enjoyment; having a VA helped to reduce simulation sickness (e.g., dizziness) (IJsselsteijn et al., 2006). In sum, the VR environment itself can be enjoyable (IJsselsteijn et al., 2006) or threatening (Meehan et al., 2002), which can influence arousal, but specifically a VA's behavior [e.g., eye gaze (Garau et al., 2005) and negative message (Lucas et al., 2018)] influences HR when people feel negatively evaluated, perhaps because people expect VAs to be less judgmental than humans (Lucas et al., 2014).

In interpersonal conflicts, negative evaluations can be accompanied by aggressive behavior (e.g., angry outbursts). The same aggressive act of a VA and a human was found to increase physiological arousal (i.e., increase in HR and EDA), but an aggressive *person* caused greater physiological signs of alarm compared to a VA (Blankendaal et al., 2015). Interestingly, when a person became aggressive, people's HR decreased substantially before it went up, but not with the VA; the EDA trend was the same for both conditions (i.e., upward after the aggressive act) though to a stronger degree with a person (Blankendaal et al., 2015). While EDA reflects general stressfulness of a VR scene (Blankendaal et al., 2015; Bosse et al., 2018), HR may be more sensitive to particular events (Meehan et al., 2002; Garau et al., 2005; Blankendaal et al., 2015), specifically VAs' social behaviors (Garau et al., 2005).

### 2.4. How Can Virtual Agents Help Train Professionals?

Studying social interactions with virtual agents is an ongoing area of active research (Bombari et al., 2015). For example, research has been done on prosocial tendencies and in group and out group biases with virtual agents (Gillath et al., 2008; D'Errico et al., 2020), and how to mitigate racial bias with VR (Peck et al., 2013; Banakou et al., 2016). One-to-many type of social conflicts, like public speaking, and how to ameliorate potential anxiety in such situations have also been covered (Slater et al., 1999; Anderson et al., 2005). However, research has not sufficiently grappled with *dyadic* interactions with virtual agents and conflicts therein. An exception is virtual interviewing situations (Villani et al., 2012; Bruijnes et al., 2015; Jin et al., 2019). As a specific type of social conflict situations, one-on-one interactions depend a lot on a virtual agent's performance and how human interactants view it.

VAs' body postures, gestures, and expressions can be designed to convincingly communicate *who* they are, for non-verbal signals illustrate different character traits for training purposes. For instance, virtual suspects for police officers' interrogation training can act dominantly or submissively (Bruijnes et al., 2015) and virtual interviewers for job seeking veterans can be friendly or rude (e.g., based on questions like “did you kill anybody?” that veterans have received in real-life) (Hartholt et al., 2019; Rosenbluth, 2019). VAs can thus take on certain character traits to influence human interactants' behavior toward them.

Recent works involving virtual agents emphasize *who* the virtual agent (VA) portrays itself to be while interacting with people in various work settings, such as in health care and education domains. VAs are treated similarly to humans in some ways. For instance, nurses had equal difficulties speaking up against real and virtual surgeons (Robb et al., 2015), which echoes how nurses are more stressed about conflicts with doctors than with each other (Hillhouse and Adler, 1997). In other cases, VAs are taken to be dissimilar to people. When VAs were interviewers in therapy contexts (DeVault et al., 2014), people shared more sensitive information with VAs than with human professionals; patients felt that VAs as mere machines were less judgmental than people, which reduced their fear of self-disclosure (Lucas et al., 2014). Whether VAs are interpreted to be more human-like or machine-like by individuals can shape people's stance toward them, leading to divergent interaction outcomes.

A VA's perceived role depends on its conversational content in dyadic interactions. Thus, a VA's conversational realism can mimic common social conflicts, as seen with aforementioned virtual interviewer that asked veterans rude questions to prepare them for real-life job interviews (Hartholt et al., 2019; Rosenbluth, 2019). In a similar vein, a conflict management VA for educators can be helpful, especially considering that interpersonal conflicts at work are common and cause stress (Bolger et al., 1989; Sliter et al., 2011), which can result in work disability or persistent psychological stress when not managed well (Appelberg et al., 1996). But to design a conflict management VA, how it delivers social conflicts via interpersonal interactions and what effects this has on people should be examined first.

### 2.5. Research Aims and Method

Our research on training educators on social conflicts in VR conceptually combines former works on VR training and research on physiological reactions in social interactions, which we specified to educators. Virtual agents can read physiological signals and adapt their behavior accordingly, but *why* and *for whom* agents should do so must first be considered per context. We thus aimed to explore (1) if and how VAs in educational settings should be introduced for conflict management training, (2) how such agents may affect educators they interact with physiologically and psychologically, and (3) what design features of an agent are important for it to handle complex socio-cultural conversations on work-related conflicts, as identified by educators themselves.

Our mixed methods research question explored is as follows. *How do University educators experience a virtual agent that brings up a potentially stressful conversation about a grading conflict as raised by a (fictional) student and would such experience be appropriate for training purposes?* We additionally measured participants' stress reactions in a quantitative measure, namely their heart rate (HR), heart rate variability (HRV), and electrodermal (SCL and SCR) responses. This was corroborated with participants' verbal and non-verbal responses to the virtual agent during the conversation itself.

Including quantitative (physiological measurements) and qualitative (behavioral and verbal reactions during the interaction and exit-interviews) data constitutes mixed-methods approach with concurrent triangulation (Patton, 1999; Creswell and Creswell, 2003; Creswell et al., 2008). Triangulation is necessary considering that mapping physiological measures to psychological processes is not clear-cut, often with many unaccounted variables (Cacioppo et al., 2007; Koruth et al., 2015). Physiological responsiveness is highly individualized (Allanson and Fairclough, 2004; Cacioppo et al., 2007; O'Brien and Lebow, 2013), and is best contextualized by looking into concurrent behavior, such as non-verbal signals like gestures or speech patterns, and accompanying verbal responses.

So we analyzed participants' interactions on *how* they talked and reacted. Also with exit-interviews, our qualitative analysis builds a rich picture of participants' experience, as well as their thinking on if and how educators will interact with a conflict management VA in the future. The theoretical underpinning is aligned with the “lived experience” approach (Giorgi, 2012); we prioritized the first-person perspective that includes implicative gestures, intonations in voice, physiological processes, and participants' own reflections. Each type of data informed other sources.

## 3. Research Design and Materials

To create a conflict conversation as described in the research question, we used an embodied conversational agent controlled through using the Wizard-of-Oz (WoZ, Dahlbäck et al., 1993) method, meaning there is a set of agent utterances that can be triggered—unbeknownst to the participant—by the researcher to maintain a conversation (more details on the apparatus and procedure are described in the following section).

The study features one within-subject variable, the *Stage* of the conversation. Stage had three levels, *small-talk, teaching*, and *conflict*. In small-talk, participants were asked casual questions, in *teaching* questions were regarding the participant's experience as a teaching professional. Finally, in conflict, a grade dispute was addressed. For purposes of the physiological signals, a breathing exercise was included, which is treated as a fourth level in some of the analyses. See section 3.3 for more details on the procedure.

To preserve conversational naturalness, we did not counter-balance conversation stages. Our qualitative data on what people said to the agent would not be meaningful with counter-balanced stages. This is a limitation to our quantitative results, which is why triangulation of quantitative and qualitative data was thus important.

### 3.1. Apparatus

**Figure 1:**
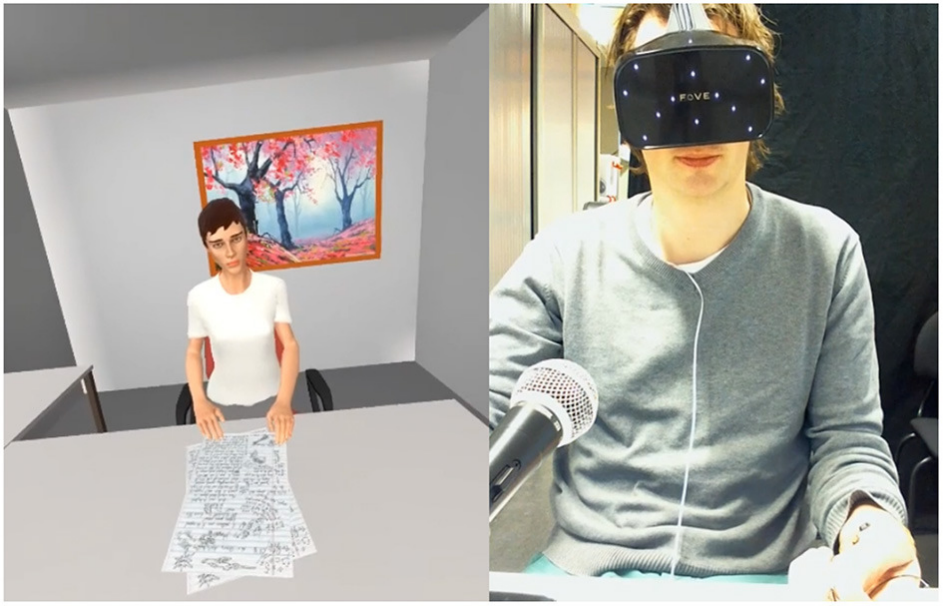
Experiment setup with the agent (left) and the participant (right).

A virtual environment was built using Unity. We did not prioritize hyper-realism in the environment itself, but kept it minimal to allow people to focus on the conversation. The environment and the VA.^1^ Participants wore the Fove VR-headset to access the virtual environment, where they were seated across the VA (see Figure 1). The VA's animation was driven by a state of the art behavior realizer (Van Welbergen et al., 2014; Kolkmeier et al., 2017a). The VA used no hand gestures and kept a neutral facial expression throughout the interaction, meaning not overly positive or negative in behavior to focus on the content of the conversation as the driver of social conflict. Speech was realized using MaryTTS.^2^ The utterances used by the VA were pre-defined and could be triggered by the experimenters (see procedure in section 3.3). A Logitech webcam recorded video and audio of the participants from a frontal perspective. This FOVE headset also captured eye-tracking data. For physiological measurements of heart rate and electrodermal activity, the BioSemi ActiveTwo system was used.

### 3.2. Participants

After receiving ethical clearance, we recruited 42 participants from University of Twente (*n* = 20) and Eindhoven University of Technology (*n* = 22) through snowball sampling by mainly targeting Ph.D. students with teaching duties. Their average age was 31 and 12 were women. Our sample was culturally diverse, with 22 out of 42 participants originating from outside of the Netherlands where the research took place. Similarly, first and second authors are not citizens of the Netherlands, and have different ethnic and cultural origins from each other. As such, our backgrounds contribute to understanding culturally sensitive factors that participants shared. As for supervision or grading related conflict with a former student, six participants explicitly remarked on such a conflict through exit-interviews or while conversing with the VA. To emphasize, we did not recruit based on their prior experience with grading conflict, and did not reveal this aspect of our experiment beforehand.

### 3.3. Procedure and Data Collection

Before the study, participants read and signed the informed consent form that stated that they will talk about students with a VA and that their participation is voluntary. When participants were directed to the study set-up, they put on the headset and sensors for the physiology measurements. Next, participants were given instructions that the VA will begin the discussion after a breathing exercise. After the first breathing exercise of 120 s, researchers *wizarded* the VA utterances from a separated area. For each *Stage* of the conversation there was a different set of predefined utterances (see Table 1). First *small-talk*, then *teaching*, and finally the *conflict* stage. Here, the VA asked for permission to talk about an anonymous, former student who voiced a complaint. The VA claimed that the student was unhappy about the received grade and blamed the participant. The wizard was not allowed to use utterances from an earlier stage. Utterances within a stage were selected in a semi-structured way. Each stage was sustained for 2–3 min each. On average, the first stage ended up shorter than the other two (Small Talk *M*= 112 s, *SD*= 30 s; Teaching *M*= 187 s, *SD*= 54 s; Conflict *M*= 199 s, *SD*= 54 s). In addition to the stage specific utterances, there were some generic utterances like “why is that?” “yes,” or “no” to drive the conversational flow. All utterances were logged with a time stamp. In the analysis, stages were segmented based on the first use of an utterance specific to each stage.

**Table 1:** Sample utterances used in the different stages of the conversation.

**Stage**	**Utterances**
Small talk	What are you currently working on?; Could you describe a typical work day?
Teaching	Have you given lectures?; Can you tell me more about a recent student project?; How do you feel about being in a teaching position?
Conflict	According to the student you did not give timely feedback; Have you had such complaints from students before?

The VA ended the discussion by thanking participants, communicating that the researcher will be back after a breathing exercise, and saying good-bye. Afterwards, all equipment for audio, video, VR, and physiological data were removed. We also assured participants that no actual students' complaints were involved in the study. Next, participants completed a questionnaire on demographics, their subjective stress levels for the past few days (Suzuki, 1997; Hashimoto et al., 2012), items on the perceived warmth and competence of the agent (e.g., *I thought the dialog partner was approachable*, Guadagno et al., 2007; Huisman et al., 2014; Kolkmeier et al., 2016). We also asked if they found the conversation with the VA stressful and if they would find similar conversations with an actual University exam board or a student stressful [modified scale (Mezo et al., 2005)]. They then partook in semi-structured interviews on their teaching philosophy, how they felt about the VA and the conversation, the VA's negative assessment about them, and what they thought about the VA for training purposes.

### 3.4. Analysis

We looked into potential outliers, failed recordings, and artifacts first. No participant indicated heart related problems and the sample did not report to be highly stressed overall; the average subjective stress level was at 0.72 on a 4-point scale from 0 to 3 (*SD* = 0.46, *MIN* = 0.00, *MAX* = 1.56) according to the stress response scale (Suzuki, 1997). From the total sample (*n* = 42), eight were excluded from physiological analyses due to failed ECG recordings, likely due to conductivity failure of the electrodes, as manifested in either completely (*n* = 5) or partially (*n* = 3) missing data and prolonged sections showing artifacts.^3^ From the remaining sample (*n* = 34), two more participants where excluded from EDA analyses (SCL and SCR) for the same reason; failure of the skin conductance electrodes. We have audio recordings from 28 post-study interviews that were transcribed; one participant did not partake in the interview and recordings were not successful for the rest. When relevant, interview notes for all participants were referred to for analysis, alongside videos.

**Figure 2:**
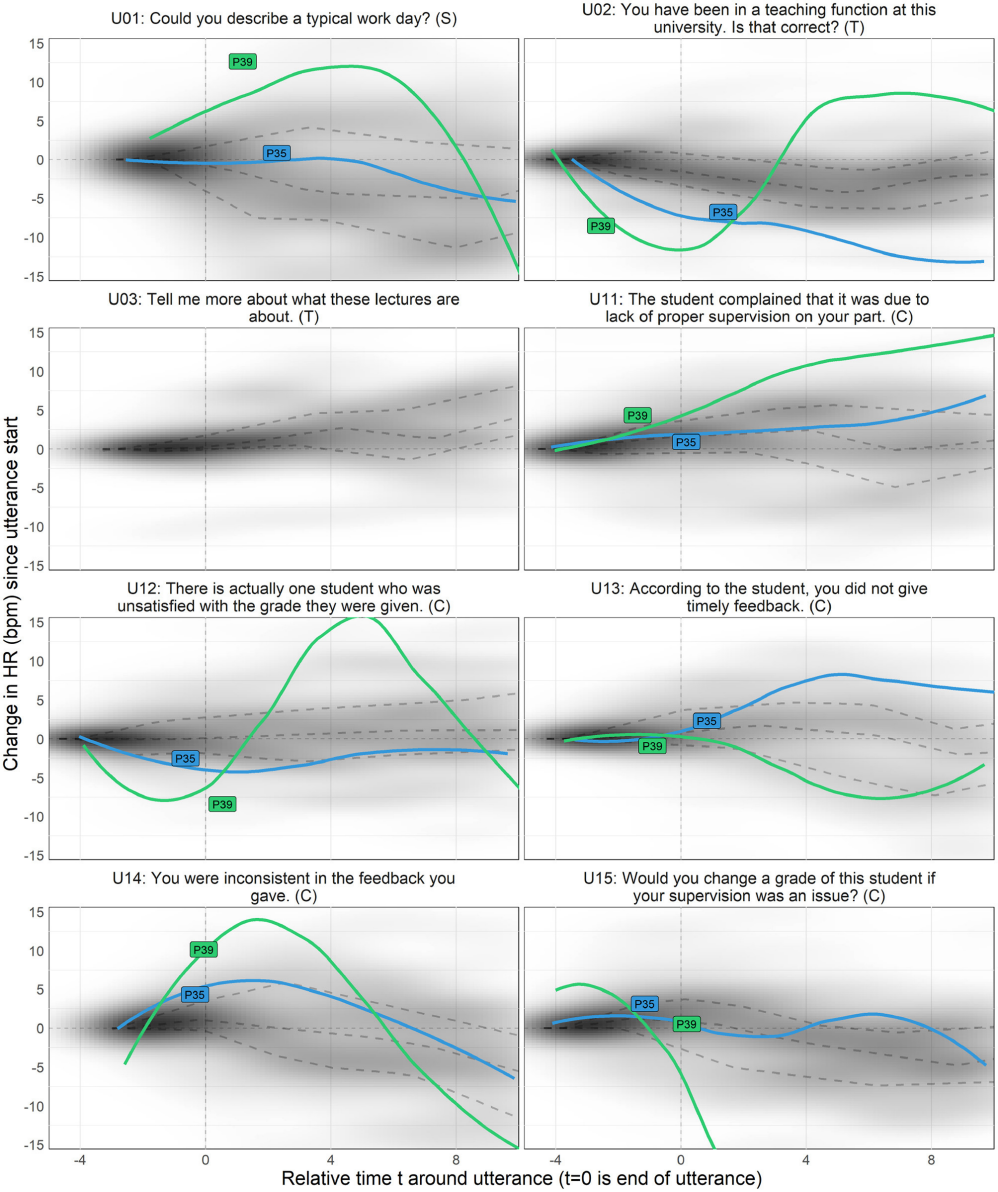
Density of changes in HR after hearing agent utterances over time, in seconds, per utterance. Utterance end-times are aligned at *x* = 0. Quantile regression lines are shown as gray dashed lines, at 75, 50, and 25% from top to bottom (RQSS method, Koenker, 2019). For illustrative purposes, trajectories of two individually examined participants are shown.

Quantitatively, we looked into our physiological data in three ways, (1) group level changes per stage of the conversation (Cacioppo et al., 2007) (Table 2), complemented by (2) individual level differences in responses on a per-utterance level (Figure 2), and (3) we explored correlations with post-experiment survey data and the physiological measures. For qualitative data, we transcribed exit-interviews and conversations with the VA, and watched videos of the interaction. We thus textually and visually coded for meaning units, i.e., when there is a change in meaning or tone in participants' spoken speech, behavior, or actions (“lived experience” approach; Giorgi, 2012), followed by grouping them for thematic analysis, which flexibly allows for divergent theoretical leanings (Braun and Clarke, 2006). The research team compared and contrasted emerging themes via multiple discussions, and allowed our quantitative and qualitative analyses to inform each other.

**Table 2:** Pairwise comparison of physio measures by stage (Tukey adjusted *P*-values).

**EMMeans**	**Cntrst**	**Est**.	**SE**	**df**	***t*-Ratio**	***P*-value**
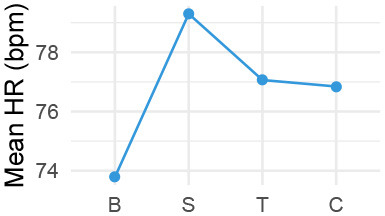	B–S	−5.51	0.93	99	−5.96	<0.0001
	B–T	−3.28	0.93	99	−3.54	0.0033
	B–C	−3.06	0.93	99	−3.31	0.0071
	S–T	2.24	0.93	99	2.41	0.08
	S–C	2.45	0.93	99	2.65	0.045
	T–C	0.22	0.93	99	0.24	1.0
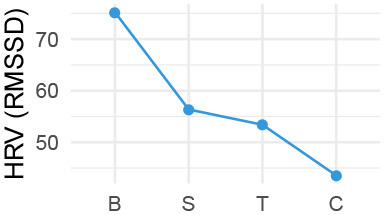	B–S	18.86	9.73	99	1.94	0.22
	B–T	21.83	9.73	99	2.24	0.12
	B–C	31.66	9.73	99	3.26	0.0083
	S–T	2.96	9.73	99	0.31	0.99
	S–C	12.80	9.73	99	1.32	0.56
	T–C	9.83	9.73	99	1.01	0.74
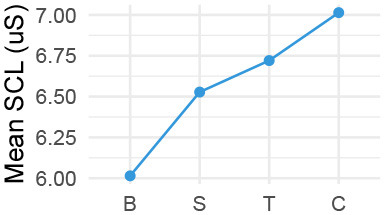	B–S	−0.513	0.116	93	−4.44	0.0001
	B–T	−0.706	0.116	93	−6.11	<0.0001
	B–C	−1.000	0.116	93	−8.65	<0.0001
	S–T	−0.193	0.116	93	−1.67	0.35
	S–C	−0.487	0.116	93	−4.21	0.0003
	T–C	−0.296	0.116	93	−2.54	0.06
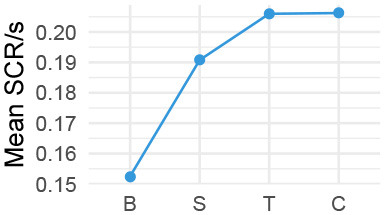	B–S	−0.038	0.009	93	−4.16	0.0004
	B–T	−0.053	0.009	93	−5.81	<0.0001
	B–C	−0.054	0.009	93	−5.84	<0.0001
	S–T	−0.015	0.009	93	−1.65	0.35
	S–C	−0.016	0.009	93	−1.68	0.34
	T–C	−0.000	0.009	93	−0.03	1.00

*The four stages are: B, Baseline; S, Small Talk; T, Teaching; and C, Conflict. Estimated marginal means from the models are in the first column*.

## 4. Findings

### 4.1. Quantitative Results

We observed a significant effect of stage for all physiological measures according to within-subjects repeated measures analyses of variance, i.e., ANOVA, HR: *F*_(3, 33)_ = 11.99, *p* < 0.001; HRV: *F*_(3, 33)_ = 3.71, *p* = 0.014; SCL: *F*_(3, 33)_ = 26.45, *p* < 0.001; SCR: *F*_(3, 33)_ = 15.13, *p* < 0.001 (individual level differences are nested within-stage level analysis). Pairwise comparisons then showed that for most measures, the difference is between the baseline and each respective stage (Table 2). Only for HR and SCL there were significant differences between the other stages. HR *decreased* significantly between Small Talk and Conflict by an estimated 2.45 beats per minute (*SE* = 0.93*bpm, p* = 0.045), although also the difference manifested for the most part between Small Talk and Teaching near significance (est. decrease of 2.45*bpm*; *p* = 0.085). SCL *increased* significantly by an estimated 0.487 μS (MicroSiemens) between small talk and Conflict (*SE* = 0.156 μS, *p* = 0.0003).

### 4.2. Exploratory Analysis

To investigate the variance in our physiological measures, we explored correlations with perception of the agent and interaction, and demographic data. To this end, we averaged the physiological measures across all stages and normalized them by the baseline measurements from the breathing exercise.

Highest correlations between the normalized physiological measures and subjective measures were found between HRV and the perceived level of the VA's autonomy. This negative correlation showed a trend toward significance (*r* = −0.35, *p* = 0.058); those with higher normalized HRV reported they believed the VA was controlled by a human (instead of by a computer). Normalized SCL moderately negatively correlated with the extent to which participants reported that such a situation would be stressful to them in a real encounter, again trending toward significance (*r* = −0.32, *p* = 0.085). The normalized HR was weakly linked to participants' self-report of finding the conversation with VA to be stressful, but was not statistically significant (*r* = 0.29, *p* = 0.12). Other self-report and demographics items, such as gender or perceived interpersonal attitudes, such as agent perceived warmth did not correlate with the normalized physiological measures.

Outside of correlations between physiological and subjective measures, we have also taken a look at correlations between demographics and subjective measures. This revealed a significant moderate correlation between the culture (*non-Dutch* coded as high) and perceived competence of the VA. Local (Dutch) participants reported low perceived competence of the VA (*r* = 0.40, *p* = 0.03). There were two non-significant, moderate correlations. First, between gender (*male* being coded as high) and the perceived level of the VA's autonomy (*r* = 0.30, *p* = 0.10), second between culture and the extent to which participants reported that this particular encounter was perceived as stressful (*r* = −0.30, *p* = 0.11 on if Dutch vs. non-Dutch participants found it to be more stressful). There were no other correlations between demographics and subjective measures.

#### 4.2.1. Descriptive Analysis

While above analyses are standard for reporting physiological measures, they serve a summative purpose. We dissected further by utterance level, i.e., *how* people were affected by different types of statements in all stages. Thus, to understand trends in our quantitative data, we visualized HR responses over time as 2D density estimate plots from all participants' response trajectories (Figure 2). Darker areas indicate that responses of many participants followed a similar trajectory of change. In addition, two participants' trajectory lines are plotted to illustrate individual differences. In selecting utterances, we ranked participants' physiological sensitivity by the greatest change in HR data from the start of the VA's utterances up to 10 s afterwards (longer time would include the next statement). No utterances shorter than 2 s were included (e.g., “yes”). Not all utterances were used for all participants, e.g., U03 was not used with either one of the two highlighted participants, to preserve conversational naturalness. This means that due to some participants' engagement level, questions like “tell me more about what these lectures are about” (U03) were not needed. To make all stages comparable between participants, the VA asked further relevant questions per stage if participants were less verbose.

Three related trends are shown in Figure 2: (1) HR peaks occurred at different points in time, in that some peaks were when people are answering, but other peaks began forming while the VA was still talking [e.g., “according to the student, you did not give timely feedback” (U13)]. (2) Each statement was associated with different patterns of HR increase and decrease for participants. An emotionally loaded stimulus can result in slower HR due to people redirecting their attention on the new stimuli or thinking process (Lang et al., 1993; Koruth et al., 2015) and some emotions decelerate the HR [e.g., suspense or fear (Kreibig, 2010)]. Accordingly, (3) some statements outside of Grading Conflict elicited high HR changes [e.g., “could you describe a typical work day?” (U01) for P35]. Each participant may be triggered by different statements due to their particular backgrounds regardless of topics covered per stage, resulting in varied physiological signatures. We hence ranked all participant responses to utterances by the greatest change in HR to further inspect why some individual variations occurred and proceeded to watch associated videos.

### 4.3. Qualitative Findings

#### 4.3.1. Video Analysis

Four participants in particular contributed a sizeable number of the highly ranked utterances (40/112 utterances = 35.7%): P24, 32, 39, and 40 (P39 is in Figure 2). P24 did not experience technical issues or discomfort, and denied the VA's statement that he did not give timely feedback (i.e., “that's not possible [...] it's always difficult to keep everyone happy”). While his HR trend suggested high arousal, he recalled that he was not stressed during the interaction in the exit-interview. P40 was often holding the HMD unlike other participants and commented on her bad eyesight afterwards. Regarding the complaint on timely feedback, she also replied “that's impossible because I always give time.” For P40, high arousal could be attributed to discomfort due to the equipment, besides conversational content. P32's video showed that he could not understand the VA well for many utterances, and shared that it was his first time in VR. His remark on untimely feedback accusation was “that's too bad. I suppose life happens.” P32 also reached out to the research team the next day that he dreamt about the same VR scene, with the VA being very negative. Limitations like the VA's speech fluency and P32's sensitivity to VR (and its novelty) could result in greater change in HR for portions of the interaction.

Interestingly, P39 said that when the conflict stage began, he felt a “twitch … Didn't really expect it, and then reacting to it was quite difficult,” and responding to the VA on untimely feedback, he said “I don't know (laughs) whether or not that was the case because we're not talking about a specific case.” Unlike others, he seemed to be more aware of his bodily or physiological changes, and his HR went steadily down until the conflict stage when it plateaued. We further checked HR rates in participants who reportedly felt physiological changes, but did not find many who seemed attuned. As another exception, P35's HR went up throughout, especially in the conflict stage and he shared, “I did feel my heart rate get faster and felt a bit uneasy sitting there. Yes, [...] it's quite real. It's like you get some negative feedback from someone important.” Looking into individual differences in participants demonstrated that physiological changes can be attributed to physical discomfort due to VR equipment, conversational disfluency of the VA, novelty effect, and/or sensitivity to the conflict scenario itself, and most could not accurately estimate their HR changes.

#### 4.3.2. Thematic Analysis

As our first theme, we found that *expecting human-likeness leads to negative evaluation*. We describe below how participants who expected a more human-like behavior from the VA negatively evaluated it for three related reasons.

***Technical limitations*. **The VA was judged on technical limitations, i.e., fluency of generated speech and appearance. Our VA and VR environment were simple, so limitations were noted by many. This caused stress in some: “I had to force myself to never miss one word from her. Maybe it's my language problem but I felt a bit stressed, and less interaction with a virtual agent” (P27). More negatively, P32 felt that it was like being “interviewed by an ATM”; difficulties communicating with the VA “massively eroded the trust” for him. P40 noted that the “environment is fictitious but [...] you notice this more because the graphics are not high end [...], which interrupts (the) conversation a lot.”

***Ineptitude for empathy*. **Participants commented about the VA's inability to be empathetic. They commented on the VA's facial expressions, e.g., “what bothers me is that she can't smile” (P41) and perceived attitude, e.g., “she asked those questions in a way that I'm more or less allergic to—‘I'm the one who knows it, and you don't know what you're talking about”' (P35). Even to the VA during the experiment, P18 said “you look a bit mad” (P18) although we only used “neutral” expressions (not overly positive or negative expressions); the VA's supposedly neutral expressions were taken to be cold or “mad” (P18). Overall, people's perception that the VA was not empathetic was considered to be a greater problem than its technical limitations. To overcome this, a personalized take on emotions displayed according to the VA's role may be preferred. P36 suggested to “make the avatar very friendly and empathic or [...] make an avatar which is much more distant (so that) you can select the avatar which under certain circumstances would be better (rather) than only one or two other choices,” suggesting a more user-configurable persona instead of a one-size-fits-all emotional performance.

***Ambivalence on “who or what am I talking to?”***People can feel comfortable vs. incredulous that a VA is engaging with them, with some needing to explicitly know *who* and how autonomous the agent is. P26 thought during the confrontation, “if it's real, what would I do? So I had a bit of a conflict there, how to respond to this person, a GIF [...] sometimes I felt that it's stupid to talk to an avatar because I wasn't sure to who I was talking” (P26). How to best respond to a confrontational “GIF” was a conflict in itself for P26, rather than the topic of the confrontation. The ambivalence on the system behind the persona and how the conversation was driven mattered to P32: “I have no information about whether my answers are simply being recorded or whether they're actually influencing the next question; whether it is in fact a conversation, or just filling in of a virtual form with my transcribed responses” (P32). The unresolved disjuncture between *who* a VA is, i.e., a conversational being that does not use pre-set responses, compared to *what* it may be, i.e., a talking virtual Q&A form, created uncertainty about the interaction itself.

As our second theme, we elaborate on considerations for immersion with the VA. *Immersion refers to situational or conversational realism, which can be de-coupled from how “real” an environment or an agent looks*. Some participants were more positive when they expected the VA to be purely machine-like. These participants focused on the fact that the VA could have human-like conversations, though it neither talked nor looked like a real human. Our subthemes are below.

***Opening up because the VA is not real*. **There were participants who were more open to discussing a sensitive topic with a VA than with a person. VAs “are virtual so I'm more open to talk about anything. But based on the assumption that they are not real” (P25). Feeling comfortable with a VA experientially was based on the pre-conceived belief that VR is not “real life.” More positively put, P28 stated “I loved it. I think it's super nice. I felt really good because [...] it is just not realistic, so you're not in the real world [...] It feels like that you can talk more about it. You feel more relaxed to talk more because it's fake. Not the conversation, I mean the whole situation can be like in a dream [...].” These participants positively viewed conversations with a machine-like VA in a virtual world as an opportunity to open up. Thus, some were more forgiving of technical limitations and focused the VA's capabilities, such as the content of the conversation itself.

***Conflict situation feels real— “put on the spot”*. **VR experiences that allow for immersive reflections about work conflicts are novel and challenging. Depending on actual teaching experience, participants could refer to conflicts with students. So some participants brought up past events to the VA: “there was a student group that was unsatisfied with their grade we gave indeed. [...] um, we have a, it was a sort of misinterpretation or miscommunication [...] between the supervision team because I didn't supervise them all by myself” (P19). Sometimes in a defensive manner, participants with prior experience of grading conflict provided more explanation of what happened, like referring to other responsible staff members. They may resort to evasive responses to the VA because of negative emotions that come with accusations and conflict. P25 said “if it's really my fault I will feel ashamed” and told the VA that “students were not satisfied with the grading; they always want more,” directing the attention away from himself.

***The problem becomes real*. **The VA's perceived unempathetic behavior is tied to the discomfort felt in the conflict situation. P25 said in the interview, “I'm not sure whether “stressful” is the right word, but at least I feel very concentrated because I have to think back. [...] the expression of the VA is not really natural so she almost never smiled, so I feel very uncomfortable when she asked questions. It feels like she's trying to blame me for something [...] like I'm the person who already did a lot of wrong things. It's like policeman, questioning me.” Similarly, P03 recalled the transition to the confrontational talk: “even if you know it's a hypothetical situation, you don't want to be put on a spot like that. [...] I would feel pretty bad if the student did not find the grade fair, even in a hypothetical situation.” Whether the situation is hypothetical or real, people's negative feelings are real when they are “put on the spot.” For instance, in that moment, P37 explained: “(I) got a bit anxious, caught off guard.” Interestingly for P1, realistic confrontations that one can face at work in VR defined immersion. “At first it was hard to take the VA seriously. You're really out of your context. At the same time when the VA raises questions that some people are not happy with you, then the problem becomes more realistic. So all of a sudden it became very immersive for me to talk with the VA because it doesn't really matter anymore (that it is virtual), and the problem becomes real and I have to pay attention and be serious.” If the conflict feels “quite real. It's like you get some negative feedback from someone important” (P35), i.e., the VA then is seen as a virtual “someone,” not just an animated agent.

As our last theme, we elaborate on *how a VA can be integrated* into real work environments. Building on the above theme on immersion we present our subthemes: a conflict management VA as a trainer was favored, but a VA as an actual conflict mediator was not favored, though this depended on cultural backgrounds of educators.

***Training educators*. **Participants thought that the VA would help for training purposes. Conflicts with students about grades do happen. As a rare incident, an experienced educator (P36) shared that once “a student was so angry about the grade I gave him, that he, uh, started throwing books. And that was very embarrassing.” Yet, the majority of our sample were Ph.D. candidates, still “learning how to supervise students” (P16). One challenging aspect for new educators is *ad hoc* responses to students: “at the beginning, one of the things that I found difficult was that you have to have a response on the spot. [...] It (VR) might be useful for that, to practice that. If you get a question like this, how might you respond to that, instead of it feeling like, ‘oh now I have to immediately respond to this thing and I have no idea what to do”' (P33). P2 similarly noted that training would be beneficial because either with students or exam committees, “I wouldn't know what would be an appropriate response (and) [...] I wouldn't know how to respond so quickly.” An added benefit of VR is that, compared to a human, a VA could induce less defensive responses since “it's more consistent. [...] with a person.at the moment (one might respond) ‘I didn't say that”' (P7). A training system “would be valuable because [...] sometimes you can be very defensive [...] Having this kind of training makes you feel aware of your feelings and then in real life, you would be more conscious, like ‘okay, this is now out of anger”' (P28). Hence, VR may be helpful since “it's also difficult to prepare yourself with another person, but (when) you feel this kind of simulation, then you're really living, experiencing that and now you can use this experience in real life” (P30). For novice teachers, VR is useful for future challenges, such as difficult conversations with students about their grades.

***Agent as mediator*. **The general consensus was the need for a human-in-the-loop when making decisions. Participants were concerned over how much decision-making power the VA and its corresponding AI system would have. Collecting and disseminating information between parties would be acceptable and could save time according to P4. If “students talk to the machine and in the end [...] summarizes it to me, perfect, I would trust the machine,” but it would be problematic if the “machine will review you and machine basically says it's your fault, so the University sends a complaint. [...] I wouldn't like to be judged because it's a machine” (P04). Others did not see a need for it. For instance, P06 stated “I don't see this as something that will help compared to things that are already there.” More critically, P36 thought that “it's the beginning of corruption” when a University institutionalizes such a system rather than trusting people, i.e., “you should only do the things like this when they are based on trust because then they can be very helpful.” In all, a VA that replaces human-to-human mediation was not favored, but one that complements existing mediation processes could be fitting as long as the VA attempts to help rather than judge educators: “tech can help, even if it's harsh, to open discussions” (P06).

***Cross-cultural differences*. **Our participants communicated about how cultural perspectives are crucial, though we did not set out to investigate cross-cultural differences at the start. We had many international or non-local participants; they commented more on hierarchical barriers “students don't have the courage to report problems. [...] I come from an Asian educational background so students have trouble talking to their teachers [...] (and) for human resources, it's usually not usually easy to deliver a negative opinion of a student. So a virtual agent makes it quite neutral” (P1). P37 declared that to fit his culture back home, the VA's “small talk would be longer [...] (and a) more indirect way of saying that” students were dissatisfied would be recommended to “soften” the message. The VA is neutral compared to educators and students who are often sensitive to power dynamics, but the VA's conversational style should still match the culture it operates in.

If the aim is to prepare foreign teachers to adapt to the Netherlands, the VA “could have been a bit more threatening” (P29); openly giving positive or negative feedback is a part of the Dutch culture. Since P29 trusts the Netherlands's larger educational structure compared to back home, if the “VA's part of the (educational) system, [...] it felt kind of safe.” Yet participants from the Netherlands had a different view. They would prefer face-to-face conversations and hoped that students would feel comfortable speaking up (if not with them with another educator). P33 shared that “if a student isn't happy with me, I really hope that they would talk to me about it, but I can imagine that sometimes they might feel weird about that, or they might feel like it would reflect negatively on them. And (then a VA) [...] could maybe take the edge off for a student.”

Educators from the Netherlands working with students from different cultures provided another facet. P19 told the VA about a struggle he had as a supervisor with “a student from a foreign culture … he was very … he was not very self-independent. We tried to make him more independent. But maybe a bit too extreme. We could have been a bit more supportive of him, support him more with the tasks he had to do for his internship.” While participants from the Netherlands would allow for a VA, it would not be preferred over real-life conversations. But for those who are not from the Netherlands (instructors or students), the VA may be preferred when considering their culture and accustomed power structure.

## 5. Discussion and Design Implications

Our divergent data sources provided a multi-faceted view on interacting with a VA about a student-teacher conflict. We first address our physiological data. While there were main effects of the conversation stage on physiological measures, we should be careful when interpreting them due to the lack of counterbalancing in our study design. In particular, SCL is a measure that is known to drift upwards over time (Boucsein, 2012). Trends in HR and SCR measures further put both the Teaching and Conflict stages at very similar levels (albeit different from Small Talk).

The exploratory follow-up analysis showed some trends for correlations with subjective measures when looking at physiological levels over the entire interaction instead of per stage. First, we note the correlation between HRV and the perceived autonomy of the agent, which was unexpected. One explanation can be that people who believed that the agent was controlled by a human, such as the experimenter or perhaps someone from the exam committee, could trigger them to feel observed, influencing people's HRV. A more expected result was the positive correlation between normalized HR and how stressful the interaction was perceived. Interestingly, this is not in line with the decrease in overall heart rate toward the end of the interaction, further suggesting that order effects may be at play.

There was an inverse correlation between SCL and how stressful participants would find this situation in a real encounter. While this is difficult to interpret, one reason could be an interaction with a virtual agent does not compare to the intensity of people's prior memory (if any) of a similar, real-life conflict. People's prior life experiences color how they view the agent and also how they may physiologically respond.

Given that HR more so than skin conductance is sensitive to socially responsive VAs (Meehan et al., 2002; Garau et al., 2005), we visually looked at HR data per utterance as descriptive analysis (Figure 2). This showed that conflicts did not always or immediately bring about a “fight-or-flight” response and that physiological signals of stress are idiosyncratic (Lazarus, 1993; Cacioppo et al., 2007; Denson et al., 2009). We may need a more nuanced view on people's physiological responses to virtual agents, especially during social conflicts, which can be helped by qualitative data.

Our qualitative results indicated that many felt judged [e.g., received “blame” (P25)], perceived the VA to be angry [e.g., “you look a bit mad” (P18)], or described the VA as not very friendly [e.g., “she can't smile” (P41)]. Being judged harshly, even if it's only virtual, can make people “feel bad” (P3) or “anxious” (P37) when there is threat-to-self. This can lead to either fact-based denial or “defensive” (P28) attitudes [i.e., the accusation was too “abstract” (P39) or “not possible” (P24)]. Thus, negative experiences were connected to *who* the VA was framed as [e.g., a know-it-all personality that “I'm more or less allergic to” (P35)] or in the ambivalence on the VA's role [e.g., “I had a bit of a conflict (on) how to respond to this person, a GIF” (P41)]. Yet, the fact that a VA is not real provided others with an opportunity to talk openly (P28). Many participants were able to accept a “hypothetical” (P03) criticism from an imaginary student as told by a VA. Further, confronting real-world problems in a virtual world can define immersion (P01); realism is not solely defined by realistic speech or image processing, but by how realistic a conversation is [i.e., “the problem becomes real and I have to pay attention” (P01)]. As corroborated with physiological data, emotions are influenced even if a VR social conflict is merely hypothetical.

Conflicts at work are stressful and damaging for health long term (Appelberg et al., 1996; De Dreu et al., 2004). But hearing out others' criticisms, or at least entertaining the possibility that one might be at fault, is at the heart of many interpersonal conflicts that a VA can help with as a reflective process. Most participants thought that such VR training would help. A VA as a mediator would be more suited in certain cultures, predominantly in Asia, where shame may be a more common feeling following blame [ “if it's really my fault I will feel ashamed” (P25)]. Shame renders the self as inadequate and isolated, while guilt focuses on one's actions, not oneself; guilt over wrongdoing is more common in Western cultures than shame (Tangney et al., 2007). Perhaps this is why interpersonal conflicts were more distressing for the Japanese than Americans (Hashimoto et al., 2012). Then, receiving criticism from a machine may be preferred: “a VA makes it quite neutral” (P1). Participants from the Netherlands wanted transparency between teachers and students and a few would accept a virtual conflict mediator only if it helps students to open up or “take the edge off” (P33). Based on our findings, we now present design implications for a conflict management VA below.

### 5.1. Give the VA a Clear Identity and Empathetic Character

Due to the nature of our study, we created a generalizable scenario and used neutral expressions for our VA. Our VA was neither aggressive (as in Blankendaal et al., 2015; Bosse et al., 2018) nor overly friendly or happy. Yet, agents may come across as cold and judgmental even in the neutral state, especially when it comes to sensitive topics like student-teacher conflicts. Thus, an agent's dialogs and behavior should convey greater empathy. We aimed for consistency with our conversation stages due to our study design, but conversationally adaptive agents that respond to verbal and non-verbal signals of users' states (e.g., reacting to perceived joy or sadness), can improve a VA's display of empathy.

### 5.2. Design for Interpersonally Varied Stress Signs With Scenarios That Fit the Job Sector

A VA designed to detect and appropriately react to people's stress during conflict management can be more empathetic. This is important since everyday stressors from teaching tasks can compound to physical and mental ill-being for educators, especially when they are new to the job (Taris et al., 2001). Disputes over grades are common, but more acute and infrequent scenarios may be added. For example, a scenario like having an angry student throw books at a lecturer are unexpected, “embarrassing” (P36's experience), and difficult to handle. Systems should realize that each individual appraises and reacts to stressors differently (Lazarus et al., 1985; Lazarus, 2006). Some people are more attuned to their physiological changes (like P39) though most people are not. Also, one's perception of being stressed is not akin to trends like HR increase (P24), and one may even feel stressed without physiological changes. Further, critical remarks from a VA can actually decrease HR (Figure 2), which happens when people feel fear based on perceived threat (Kreibig, 2010; Blankendaal et al., 2015). People's self-perception as well as a suite of physiological measures (e.g., HR, HRV, SCL, and SCR) can provide a better picture, best done over several interactions with a VA for within-person accuracy. Our results indicate that HR for reactions to the agent and SCL for assessing situational impact should be put together.

### 5.3. Adapt the VAs Conversational Style to Account for Culture

Rather than deploying a one-size-fits-all design, a culturally appropriate VA is recommended. Types of conflicts, how they are handled, and how they influence people involve culture as an important dimension (Hashimoto et al., 2012). Even physiological measures show minor trends in cross-cultural differences (Tsai and Levenson, 1997; Tsai et al., 2002). To match for culture, the VA can try different conversational patterns. For example, small talk can last longer and a conflict can be brought up more indirectly to soften the blow in Asian cultures (P37). Also, organizational culture and how different roles confront conflict at work should be considered; nurses for one find it more stressful to have conflicts with doctors than with other nurses (Hillhouse and Adler, 1997), to the extent that nurses had equal difficulty speaking up against a virtual and real surgeons (Robb et al., 2015). When there is a hierarchy involved, how the society and an organization itself deals with conflicts introduces interconnected cultural aspects.

Many of our non-Dutch participants remarked that students may have difficulties bringing up conflicts, but most the Netherlands educators expected students to bring up issues face-to-face, or learn to do so. Thus, the biggest clash may be when different cultural backgrounds collide. P19 from the Netherlands had a foreign student. He pushed for the student to become more independent, “but maybe a bit too extreme” and wished he had been more supportive. P29, a non-Dutch participant, recommended that the VA can be “more threatening” to help non-Dutch educators prepare for direct communication style in the Netherlands, since both positive and negative feedback are openly given by students and colleagues. Globally, we now have greater options for international education and work, so VAs for conflict management should be mindful of cultural backgrounds of people they interact with. Additionally, they can help people become aware of how their own cultural backgrounds affect others from different cultures, and provide people with ways to practice and adapt to new cultural and organizational customs.

### 5.4. Limitations and Future Work

Our sample had many Ph.D. students with teaching duties; research with experienced staff members is needed. A more rigorous study design with counterbalancing of conditions and more exact control of stimuli duration are required to be able to derive better quantitative insights from the physiological measures. In the future, other sources of social signals could be included in the measurements. For one, facial expressions are important for conveying people's opinions, comments, and conversational turn-taking (Poggi et al., 2013). While this was still a technical limitation at the time of conducting this study, recently facial expression recognition has even made it's way into consumer hardware.^4^ Lastly, we initially did not set out to explore cultural differences, but we were open to new insights that our qualitative data provided. Cross-cultural studies on people's behaviors in VR are rare, especially considering situations that are pertinent to our daily lives. One recent study suggests that there is little difference across cultures when it comes to people's evacuation behavior and crowd flow (Lin et al., 2020). However, dyadic social conflicts may show greater intercultural variations, as our research indicates. Future research should prioritize cross-cultural comparisons.

As for gathering data on physiological and emotional signals, we mention ethical concerns. In future applications, people should be made aware of how their data are collected and analyzed; they should have control over their highly personal data, such as for learning about their physiological profile or opting out. While ethical trade-offs should be researched on, a future research area can better address how a system should respond to emotional and physiological changes according to various social situations, beyond conflict training. Designing empathetic agents could include physiological pattern detection as a reason to display certain facial expressions or behaviors, rather than merely assuming that an agent that for example, smiles more in general, will be viewed as empathetic. People's verbal, behavioral, and physiological input are valuable and related data that can better cater to individuals' contexts when used in a concerted way in a system. If and how this can be achieved deserves thorough research.

## 6. Conclusion

We explored a conflict training virtual agent with University teaching staff. The focus was on educators' perception of the VA, its potential future role, and how the VA influenced them physiologically and psychologically. We triangulated data on their non-verbal and verbal behavior during the interaction with their cardiac and electrodermal response patterns. We gathered their impressions about the VA through exit-interviews. While main effects of conversation stage on physiological measures require further research with the limitations of the study design addressed, there were emerging trends in the correlation with subjective measures and the overall normalized physiological responses. For one, there was a cultural difference in judging the agent's competence: Dutch participants found the VA to be less competent than non-Dutch participants. This relates to our qualitative insight. Dutch participants wanted face-to-face conflict mediation without involving a VA, but non-Dutch participants thought a VA for conflict mediation can help people “save face” while addressing conflicts. When designing future conflict management VAs, we recommend empathetic speech and facial expressions, as well as culturally sensitive behavioral norms.

## Data Availability Statement

The raw data supporting the conclusions of this article will be made available by the authors, without undue reservation.

## Ethics Statement

The studies involving human participants were reviewed and approved by Ethical Review Board, Human-Technology Interaction, Eindhoven University of Technology. The patients/participants provided their written informed consent to participate in this study. Written informed consent was obtained from the individual(s) for the publication of any potentially identifiable images or data included in this article.

## Author Contributions

ML and JK conducted the experiment, analyzed all the data, and wrote the manuscript. WI and DH provided the facilities, materials, and edited the manuscript. All authors contributed to the article and approved the submitted version.

## Conflict of Interest

The authors declare that the research was conducted in the absence of any commercial or financial relationships that could be construed as a potential conflict of interest.

## Footnotes

^1^Created with the Unity Multipurpose Avatar system, see https://github.com/umasteeringgroup/UMA.

^2^http://mary.dfki.de/.

^3^Potential causes for these failures were experimenter error in applying the sensors or due to unexpected movements of the participants interfering with the setup.

^4^See for example https://www.vive.com/eu/accessory/facial-tracker/.
